# Perception and management of *Oestrus ovis* human myiasis by physicians: Exploratory survey in an endemic area (Italy)

**DOI:** 10.1371/journal.pone.0322904

**Published:** 2025-05-02

**Authors:** Fahad Ahmed, Carlo Carta, Daniele Satta, Luca Varcasia, L. Kirsty Pourshahidi, Sebastian Alessandro Mignacca, Lia Cavallo, Antonio Scala, Antonio Varcasia, Claudia Tamponi

**Affiliations:** 1 Nutrition Innovation Centre for Food and Health (NICHE), School of Biomedical Sciences, Ulster University, Coleraine, Northern Ireland, United Kingdom; 2 Laboratory of Parasitology, Veterinary Teaching Hospital, Department of Veterinary Medicine, University of Sassari, Sassari, Italy; 3 Opthalmologist, ASL N.1, Sassari, Italy; 4 General physician, ASL N.1, Alghero, Italy; 5 Department of Agriculture, Food, and the Marine - Pathology Division - Backweston Campus, Celbridge, - Ireland; Addis Ababa University, College of Veterinary Medicine and Agriculture, ETHIOPIA

## Abstract

**Background:**

Myiasis caused by the sheep nasal botfly is endemic in countries where sheep and goats are largely reared, while humans serve as incidental hosts. Ophthalmomyiasis in humans caused by *Oestrus ovis* is documented globally but is most prevalent in Mediterranean countries, highlighting the significance of this myiasis as a neglected disease.

**Method:**

A thorough questionnaire covering frequency of disease occurrence, seasonal patterns, and management of *O. ovis* was forwarded to clinicians in Italy gathering data from 100 respondents across diverse regional and occupational backgrounds.

**Results:**

Majority of respondents were from endemic areas of the Southern Italy and main islands, like Sardinia (52%) and Sicily (42%), with 81% representing physicians. Notably, 31% of physicians reported treating ophthalmomyiasis cases, while 80% expressed interest in further information. Seasonal trends revealed a peak during summer (χ² = 29.429, df = 4, p < 0.001), notably among outdoor workers in contact with farm animals (χ ² = 18.059, df = 2, p < 0.001). Diagnosis relied on symptoms or parasite detection, with ocular regions being the most common site of infestation. *O. ovis* was identified in 13% of cases (χ² = 20.368, df = 3, p < 0.05), with physicians emphasizing the importance of removing larvae painlessly to avoid complications. Finally, physicians reported the efficacy of mercuric oxide ointment and the use of topical povidone-iodine for ocular localization, combined with antibiotics and corticosteroids.

**Conclusions:**

Diagnostic challenges and the persistence of recurrent infestations highlight the need for enhanced disease surveillance and clinician knowledge to effectively manage and mitigate the impact of ophthalmomyiasis as it continues to emerge as a significant public health concern.

## 1.Introduction

Myiasis (from the Greek *myia*, which means fly) encompasses infestation by several dipteran larvae, including those of Calliphoridae, Oestridae, and Sarcophagidae families [1]. Myiasis by Oestridae family manifest as primary myiasis, where biophagous three larval instars feed on living tissues, or secondary myiasis, characterized by larvae feeding on dead tissues [2,3]. Myiasis caused by the sheep nasal botfly, *Oestrus ovis*, is endemic worldwide, in countries where sheep and goats are largely reared and develop in their nasal cavities and sinuses. Transmission occurs when viviparous females fly and inject larvae into the nostrils of sheep or goats [4]. Humans are incidental hosts [5], with myiasis potentially occurring in several anatomical sites, such as the eyes, nose, pharynx, stomach, and ears [6]. Eye infection in humans occurs when a female fly is attracted to the pupil, resembling a target spot or hole, striking the cornea, and laying the larvae onto the conjunctival fornix. Ophthalmomyiasis is usually self-limiting due to the non-availability of nutrients, in the human tears, for the larval development [7,8]. In more severe cases, they can cause significant infestation of the eyes, potentially leading to the need for enucleation of the ocular globe [1,7,8].

In most human cases, *O. ovis* leads to external ophthalmomyiasis, which has no specific symptoms associated with it, but the movement of hooks and spines on the larva’s body may cause conjunctivitis. The larvae are mobile and move quickly under the slit lamp. Typically, they can be seen crossing cornea and conjunctiva [4,9]. Larvae can survive in the conjunctival sac for up to ten days when left untreated [10]. If not diagnosed promptly, larvae can occasionally penetrate the sclera and move into the eye, resulting in so called ophthalmomyiasis interna, compromising the globe of the eye [6,8,11–14].

Ophthalmomyiasis caused by *O. ovis* is documented globally but it is most prevalent in Mediterranean countries and other areas where climatic conditions favor the development of dipteran flies [4,15,16]. People living or working in farming areas and those who live near small ruminant premises are particularly exposed to infestation and are typically thought to be at the most significant risk [4,17,18]. However, ophthalmomyiasis caused by *O. ovis* are recorded frequently in urban areas [19–21] and in patients who never had contact with farm animals [22]. More recently, cases in human have also been documented in tourists returning from seaside summer holidays [6,23], highlighting the significance of myiasis for travel medicine and emerging zoonosis.

A significant number of medical professionals continue to misdiagnose and underestimate ophthalmomyiasis. The infestation may be grossly underestimated because many infected people typically treat their self-using empirical therapy and homemade medications without referring to physicians [2,24]. Myiasis presents itself most commonly as a painless and only in a smaller proportion has symptoms. For humans to present early to physicians, they need to be aware and must be able to recognize symptoms through the routine practice of practicable screening.

To the authors’ knowledge, there are no other surveys highlighting these issues in people who constitute the majority at risk for disease and late presentation. This study aims to evaluate the level of awareness, knowledge, and practices among participants - including both physician and other medical, but non-physician professionals in Italy, regarding myiasis caused by *O. ovis*. Finally, it will offer insights from field and laboratory experiences of veterinarians collaborating with physicians from an endemic country, with a specific focus on management strategies.

## 2. Methods

### 2.1 Ethics statement

Ethical approval and additional details regarding participants was not sought for this study in accordance with the regulations in force (Italian Legislative Decree n. 26/2014, implementing the European Union Directive n. 2010/63/EU), as no preclinical experiments or clinical trials involving human beings were conducted.

### 2.2 Study area and population

A cross-sectional survey was employed to assess the perception, treatment practices, and risk factors associated with ophthalmomyiasis among physicians and non-physicians in Italy. In Italy, residents have the autonomy to select their own general practitioners (GPs), providing an opportunity to sample a wide and varied patient base. GPs were chosen randomly from various regions across Italy, ensuring geographic and demographic variability, which enhanced the representativeness of the sample across both urban and rural settings. GPs thus served as the primary access points to patients, allowing us to collect data from a substantial sample of individuals who had visited or received treatment from these physicians. Additionally, other participants, including farmers and veterinarians, were randomly invited to ensure representation from affected groups and varied backgrounds.

### 2.3 Questionnaire

Data collection was conducted using a self-administered questionnaire designed to capture detailed information pertinent to the study’s objectives. The questionnaire underwent a multi-stage development process, beginning with a review of existing literature on ophthalmomyiasis, including commonly reported symptoms, diagnostic methods, risk factors, and current treatment protocols. Input was also sought from public health experts and GPs to ensure the questionnaire’s relevance and clarity. To further ensure validity, the questionnaire was piloted on a small group of physicians not included in the final sample. Feedback from the pilot group was used to refine the wording and structure of questions, ensuring clarity and reducing the likelihood of incomplete responses.

The finalized questionnaire consisted of several key sections. The first section collected socio-demographic information, including age, gender, educational background, geographic location, and occupation. The subsequent section addressed disease occurrence, focusing on the presence of symptoms, duration, and frequency, with specific attention to seasonal patterns and other environmental risk factors that might influence the onset of ophthalmomyiasis. A critical section of the questionnaire focused on treatment protocols and management approaches, where participants were asked to detail any medical interventions received, including medications, surgical procedures, and supportive therapies. Physicians were specifically asked to list all prescribed medications, allowing current practices used to manage ophthalmomyiasis cases.

The survey was conducted over a 21-month period, from January 2021 to September 2022. A total of 130 questionnaires were randomly distributed across Italy, including Sardinia, with 100 completed and accepted. The questionnaire was available both online by Microsoft forms and in print, allowing participants such as farmers and veterinarians to choose their preferred method of completion. Digital distribution channels included e-mail and WhatsApp, which were effective in reaching participants quickly and efficiently, especially those who preferred completing the survey remotely. For those without access to digital platforms, printed versions were distributed in person, either during GP visits or through community outreach efforts in collaboration with healthcare workers.

To ensure the integrity of the data, all returned questionnaires were reviewed for completeness. Questionnaires with significant missing data were excluded from the analysis. Additionally, responses to open-ended questions were carefully analyzed to extract qualitative insights regarding patient experiences and disease management.

### 2.4 Statistical analysis

All data collected from the survey were compiled in an Excel® spreadsheet for Windows®. Data was coded, anonymized, validated, and analyzed using RStudio version 4.3.1 (Integrated Development Environment for R, Boston, MA). The association between variables was assessed using the chi-square test, and statistical significance was set at p < 0.05.

## 3. Results

### 3.1 Provenience and occupational profiles of the respondents

Out of 100 respondents who answered the survey, the majority were from Sardinia (52%) and Sicily (42%), with a smaller representation from Piedmont (2%) and Calabria, Campania, Emilia Romagna, and Umbria regions (1% each). Among the respondents, 80% were physicians, with family physicians or after-hours service physicians comprising the most significant subgroups (47.5%; 38/80), followed by ophthalmologists (26.2%; 21/80), and ear-nose-throat (ENT) specialists (3.8%; 3/80) and other specialists (22.5%;18/80). Veterinary doctors (n = 5), researchers (n = 2) and others such as nurses (n = 9), and non-specialists (i.e., 3 farmers), made up 20% of the non-physician respondents.

### 3.2 Physician practices, experience and their perspectives

The 80 physicians examined an average of 1,686 patients annually (range 1,000–12,000 patients) and had been practicing medicine for an average of 11.7 years (range 1–45 years). When queried about their experience with ocular, oral or nasal myiasis, 38.75% (31/80) responded affirmatively, while 47.5% (38/80) had never encountered it, and 13.75% (11/80) had never encountered it but had heard of it. Physicians that diagnosed cases had the following year of experience: 1–5 years (n = 8), 6–10 years (n = 4), 11–20 years (n = 6), over 20 years (n = 13).

Regarding the frequency of cases treated per year, responses varied with 16 physicians reporting 1 case/year or less, 7 reporting 2 cases/year, 2 reporting 3 cases/year, 4 reporting 4 cases/year, and 5 more than 5 cases/year. Among the other respondents, 4 answered they have had experience with myiasis (2 nurses, a veterinarian and a farmer). Additionally, 80% of the total respondents expressed interest in receiving more information on the topic, while 20% (13% of which were physicians) indicated no interest.

### 3.3 Seasonality and risk factors

Seasonal occurrence revealed that 54.3% (19/35) of cases were diagnosed in summer (June-August) (χ² = 29.429, df = 4, p < 0.001), 11.4% (4/35) in spring (April-May), 8.6% (3/35) in September, 22.8% (8/35) and a single case (2.9%) was observed in February. Additionally, 22.8% (8/35) of cases were observed across an extended period from late spring to late summer (May–September).

Regarding the type of patients affected, 71.4% (25/35) were individuals who worked outdoors and had contact with animals (animal species not clarified) (χ² = 41.686, df = 3, p < 0.001), 17.1% (6/35) worked outdoors but lacked animal contact, 8.6% (3/35) worked indoors but had some interaction with agricultural environment (e.g., trekking, camping, or farmhouses), and only 1 (2.9%) worked indoors and without any apparent contact with animals and/or agricultural environment.

When asked about the potential correlations with patient characteristics or risk factors observed, the respondents reported a higher occurrence in male individuals (37.1%; 13/35) than in females (5.7%; 2/35) (χ² = 27.457, df = 1, p < 0.001), suggesting that male may be at significant risk for the occurrence of myiasis. Additionally, 5.7% (2/35) hypothesized a correlation with age and infestation in a child was reported (2.9%). Furthermore, 17.1% (6/35) of respondents suspected lifestyle as a contributing factor (more details of “lifestyle” were not given), while 22.9% (8/35) reported no correlation with lifestyle. One physician interviewed suggested that males aged between 20 and 50 years and females aged between 75 and 80 years were particularly susceptible age groups. Regarding suspected source of infestation, 65.7% (23/35) attributed the infestations with countryside contact, 17.1% (6/35) mentioned presence of sheep, and, interestingly, 14.3% (5/35) reported instances originating from beaches (X² = 18.059, df = 2, p < 0.001).

### 3.5 Etiologic agents, myiasis diagnosis and its localization: medical insights

Among the 31 physicians that reported experience of myiasis the 74.2% (23/31) did not request a specific diagnosis. Notably, only in 6 cases (19.4%) morphological identification of the causative agents has been requested, while molecular biology and serological diagnosis were requested in 1 case per each.

Regarding the 35 reported cases of myiasis, *O. ovis* was identified as the etiologic agent in 37.1% (13/35) (X² = 20.368, df = 3, p < 0.05). Additionally, 11.4% (4/35) of cases were attributed to nematodes (species not clarified) with ocular site involvement, while a single case was attributed to other Diptera species. In 45.7% (16/35) of cases, the diagnosis of myiasis was confirmed based on symptoms and anamnesis, while the isolation of a parasite was carried out in 40% of cases (14/35). In a single case the answerer reported the parasite found had not been identified. Ocular localization emerged as the predominant infestation site, reported from the 74.3% (26/35) of respondent that had experience of myiasis (X² = 51, df = 3, p < 0.05). Additionally, infestations were reported as oral-pharyngeal (8.6%; 3/35), nasal or nasopharyngeal (5.7%; 2/35) and affecting the ear (5.7%; 2/35).

### 3.6 Treatment approaches and follow-up

Treatment approaches varied among the physician respondents, with 29% (9/31) reporting the initial step of manual removal of larvae as a crucial part of the treatment. 64.5% (20/31) of physicians prescribed antibiotic cover (not specified), in most cases 38.7% (12/31) in association with corticosteroid, administered as eye drops or ointment. 12.9% (4/31) physicians reported the effective use of topical povidone-iodine for ocular localization, often combined with antibiotics and corticosteroids.

Interestingly, 19.4% (6/35) of the physicians reported in the past the use and efficacy of mercuric oxide (yellow) ointment at a concentration of 2%, now commercially unavailable from last few years.

Regarding the duration of symptoms, 9.7% (3/31) of physicians reported an improvement in symptoms in 1 day only, while 45.2% (14/31) within 2–6 days, 19.3% (6/31) 7–10 days, and 9.7% (3/31) 10–21 days. Two of the physicians (6.4%) reported the resolution of symptoms soon after the larval removal. 12.9% (4/31) of physicians could not respond due to a lack of follow-up with patients. Lastly, 25.8% (8/31) of physicians reported recurring infestations in the same patients.

## 4. Discussion

This study highlights the perception and knowledge of *O. ovis* myiasis among Italian physicians in endemic regions from Southern Italy and main islands. The awareness of myiasis among Italian physicians was good, though significant proportion (47.5%) of them had never heard of it. The survey participants indicated a higher occurrence of ophthalmomyiasis in humans during the summer. This can be attributed to higher prevalence of *O. ovis* myiasis among sheep in summer in Mediterranean countries, especially in the southern regions of Italy [4,5,26,27], where Mediterranean climate offers ideal conditions for the biological cycle of various oestrids [25]. Consequently, myiasis in humans is more common during these months [6]. Additionally, climate change is unavoidable phenomenon, and higher temperature may accelerate fly growth, leading to increase in number of fly generations throughout the year [28].

Pampiglione and Giannetto [15] reported that farmers and shepherds who live close to sheep are most likely to get myiasis. This is consistent with the results of our survey, in which 65.7% of reported infestations were contracted in rural areas, and 17.1% reported the presence of sheep. Recently, there have been numerous reports of infestation in humans returning from Mediterranean seaside vacations [4,6]. This observation has also been supported by findings of respondents, who indicated that the risk of myiasis is higher in humans visiting seaside beaches. In terms of number of cases, it is reasonable to infer that the overall number of cases evaluated by physicians has increased. Pampiglione’s survey [29] of Italian physicians in the 1950s revealed that 8% (n = 414) of the questioned physicians reported having treated at least one case of *O. ovis* myiasis in their professional careers [29]. In contrast, 38.7% of the physicians (31/80) who answered our study reported having encountered myiasis at least once in their career, including 22.5% (18/80) who treated more than one case. Our study shows a higher number of observations by young physicians (1–5 years) compared to those who have been working for more years (6–10 and 11–20 years, respectively). This might be explained by an increased awareness of the disease, a higher number of infestations over the years, and/or an increase in rural tourism of the patients [6].

Like previous studies, our survey indicated that almost half of the cases were reported in rural settings, and several of them denied interaction with animals [4,6]. It is possible to assume that the rate of underreporting is significantly higher in rural areas due to self-cure patients that did not consult the healthcare facilities [30]. Especially farmers often resort to self-treatment practices using unconventional methods, such as using exhausted motor oil and tobacco smoke to cure themselves (Antonio Scala personal observation). Furthermore, there has been a recent increase in infestation rates in metropolitan settings. This indicates that the risk of infestation rises when there are no other hosts for the flies to infest besides humans [31,32]. Additionally, due to structural resemblance of the human face to small ruminant nostrils, flies perceive human eyeballs as potential targets. Consequently, numerous cases of ophthalmomyiasis, void of animal interaction or travel history have been documented [4,9,33,34].

The survey found that only 25.8% of medical professionals have sought a definitive diagnosis for ophthalmomyiasis. This demonstrates physicians’ lack of concern about the diagnosis of the disease, which supports the prevailed conviction in rural areas and aggravates underreporting. Ophthalmomyiasis will indeed run its course, but the treatment can help reduce complications. This lack of concern could be due to shepherds living far from medical facilities in rural areas, leading them to rely on traditional treatments. A survey by Pampiglione et al. [15] revealed that only 1% of shepherds seek help from a physician within the Etnea area (Sicily) [19].

The findings of our study showed that men were more infected with *O. ovis* than women. This could be due to the fact that in Italy men are primarily involved in livestock management and spend more time outdoors, making themselves more likely to interact with agricultural environment and small ruminants. Indeed, the existence of these risk factors increases the likelihood of becoming infected. On the other hand, certain aromatic chemicals, including pentanoic acid, dimethyl disulfide (DMDS), butanoic acid, and hexanoic acid, attract female botflies [35]. Therefore, use of cosmetic products or sunblock creams may lead to an individual’s risk of infestation [6,36], as it seems from some reports recently published in Corsica and Sardinia [5,6]. Nasal myiasis has been frequently reported in middle-aged and elderly patients [37]. This is consistent with our findings where physicians suggested a possible correlation between middle age and occurrence of myiasis. One physician noted that elderly patients may be more at risk which might be due to several other factors such as immune suppression, poor hygiene and chronic diseases [38].

Due to the lack of consensus on effective treatment that may lead to the death of fly larvae, several drugs are currently being used as palliative to alleviate clinical symptoms. Some have little or no impact on the pathology, diminishing the public’s belief in the physician’s ability to treat this condition. The questionnaire provides the definitive management of *O. ovis* infestation. Physicians in the survey emphasized the importance of promptly addressing symptoms by removing larvae from the eye which can be done either manually using forceps or with the use of local anesthetic agents. Additionally, physicians observed the effectiveness of povidone-iodine eye drops in localizing the infestation, along with antibiotics and corticosteroids in managing ophthalmomyiasis in all instances. This comprehensive treatment approach has been found to prevent bacterial entry and reduce the risk of secondary infestations [4,19].

### Conclusion

This study sheds light on ophthalmomyiasis, giving insights into awareness, perceptions, and management of *O. ovis*. The findings emphasize the need for education, particularly among healthcare professionals, to improve early detection and management of ophthalmomyiasis, especially given its high frequency in southern Italy and main isles where this parasite is ubiquitous and small ruminant husbandry is prominent. Data from this study can inform targeted interventions to raise awareness among clinicians and understand the real impact of disease. Due to symptoms overlapping with other ocular disorders, full eye examinations are fundamental to avoid misdiagnosis and delays in treatment. Finally, follow-up is vital to ensure treatment efficacy and monitor complications.

## Supporting information

S1 File Questionnaire submitted to enrolled physicians. (DOCX)
