# Supplementary material for: Perception and management of Oestrus ovis human myiasis by physicians: Exploratory survey in an endemic area (Italy)
Source: PLoS One. 2025 May 2;20(5):e0322904. doi: 10.1371/journal.pone.0322904 (PMC12047822; doi:10.1371/journal.pone.0322904)
Supplement: S1 File — (DOCX) [file pone.0322904.s001.docx]

# Università di Sassari, Italia

## Questionario sulla Miasi Oculare e Oronasale

Laboratorio di Parassitologia e Malattie Parassitarie, Dipartimento di Medicina Veterinaria, Università di Sassari, Italia

Approvato dal Comitato Etico: Organismo Preposto al Benessere Animali Università degli Studi di SassariCodice di Approvazione: Prot n.95822

Domande di Screening:L'Università di Sassari sta conducendo una ricerca sulla miasi oculare e oronasale. La partecipazione è volontaria e tutte le risposte saranno trattate in modo confidenziale.

Lo studio mira a valutare la frequenza delle infestazioni, identificare i fattori di rischio e approfondire i metodi di diagnosi e trattamento adottati dai professionisti sanitari.

Tempo stimato per la compilazione: 10-15 minuti.

## Criteri di Idoneità

Puoi partecipare se soddisfi i seguenti criteri:
- Hai almeno 18 anni.
- Sei un professionista sanitario con esperienza in oftalmologia, otorinolaringoiatria, medicina generale.
- Hai avuto esperienza con casi di miasi oculare e/o oronasale.

Se soddisfi i criteri, procedi con il questionario. Per domande, contattare:
Nome:
Email:

Date:

## Consenso Informato

☐ Ho letto e compreso le informazioni sullo studio e ho avuto l'opportunità di fare domande.

☐ Comprendo che la partecipazione è volontaria e che posso ritirarmi in qualsiasi momento senza fornire una motivazione.

☐ Tutti i dati saranno trattati in modo anonimo e sicuro.

☐ Dichiaro di avere più di 18 anni e di soddisfare i criteri di partecipazione.

☐ Do il mio consenso informato a partecipare allo studio.

## Sezione 1: Informazioni Sociodemografiche

## ☐ Età: _____

## ☐ Genere: ☐ Maschio ☐ Femmina ☐ Altro (specificare) _____

## ☐ Livello di istruzione:

## ☐ Professione: _____

## ☐ Da quanti anni lavori in questo campo? _____

## ☐ Quanti pazienti visita mediamente in un anno? _____

## ☐ In quale città/regione lavori principalmente? _____

## Sezione 2: Esperienza con la Miasi Oculare e Oronasale

☐ Hai mai riscontrato casi di miasi oculare e/o oronasale nei tuoi pazienti?

1. Sì 2) No

☐ E' interessato a ricevere aggiornamenti su questa ed altre parassitosi?

1. Sì 2) No

☐ Se sì, quanti casi mediamente riscontri all'anno?

☐ Dove si presume sia avvenuta l’infestazione nei casi osservati?

☐ In quale periodo dell'anno si sono verificati generalmente i casi di infestazione?

☐ Quali pazienti sono stati generalmente infettati dalla miasi oculare e/o oronasale?

## Sezione 3: Diagnosi e Trattamento

☐ Hai mai richiesto o effettuato una diagnosi specifica per miasi?

☐ Sì ☐ No

☐ Qual è l'agente eziologico riscontrato (in caso di valutazione morfologica specialistica)?

☐ Se sì, quale metodo diagnostico hai utilizzato?

☐ Dove si è verificata la localizzazione dell'infestazione?

☐ Quali trattamenti hai adottato?

☐ Hai notato una correlazione con il sesso del paziente?

1) Sesso maschile 2) Sesso femminile 3) Non ho notato correlazione

☐ Durata sintomatologia ed evenuali complicazioni

☐ Hai mai riscontrato infestazioni ripetute negli stessi pazienti?

☐ Sì ☐ No

## Sezione 4: Raccomandazioni e Considerazioni Finali

☐ Quali misure ritieni più efficaci per prevenire la miasi oculare e oronasale?

☐ Hai ulteriori commenti o suggerimenti?

Grazie per la tua partecipazione! Il tuo contributo aiuterà a migliorare la comprensione e la gestione della miasi oculare e oronasale.
